# 

**Published:** 1856-04

**Authors:** 


*To Correspondents.*—A reply to Prof. Flint on Pneumonia, by Dr. Geo. Burwell, of this city, is received too late for insertion in the present number. We shall give it place in our next.

*Physical Exploration and Diagnosis of Diseases affecting the Respiratory Organs.* By Austin Flint, M. D.

We can only acknowledge the receipt of this work in our present number. In our next we shall endeavor to devote to it that space and careful attention which its merits demand. At present, aside from the mere announcement of its publication, we can only cordially commend it to our readers.

---

Besides the above, we acknowledge the receipt from the publishers, Messrs. Blanchard and Lea, of Barlow's Practice of Medicine, Budd on Diseases of the Stomach, Lehmann's Chemical Physiology, Lehmann's Physiological Chemistry, Parrish's Practical Pharmacy, Miller's Principles of Surgery, Sargent's Minor Surgery and Neill and Smith's Compend of Medicine. We shall endeavor to accord to these as early a notice as possible.

---

We have received (since the acknowledgment above) a copy of the Report of the Health Physician of Buffalo, for the year 1855, by John Root, M. D. It comes too late for notice in this number.

Also from Phinney & Co., a copy of Budd on the Stomach, and "Digestion and its Derangements, by Dr. Chambers."

Our readers will see that we are well stocked with material for future book notices.

Messrs. A. I. Mathews & Co.'s Annual Circular, has made its appearance for the year 1856. As usual, it comes in the neatest possible form, and is really a convenience to the physician. The profession have a due appreciation of a competent and honest druggist, and, though our friend's new quarters are rather too splendid for a bashful man, they are only indicative of his inherent fondness for first-rate articles.

An addendum to the circular is a list of works on Medicine and the Collateral Sciences. We would say to many inquiring of us for books noticed in our pages, that they may procure them through Mr. Mathews as cheaply and promptly as through any other channel.

To those remitting money to us through him, (as is frequently done,) we may add that his receipt for moneys paid the Buffalo Medical Journal is, as heretofore, good against us.

---

*Annual Meeting of the American Med. Association.*—This takes place in Detroit on the first Tuesday in May next. Every regularly organized society is entitled to one delegate for every ten members. We hope that all societies will be largely represented, and that the occasion may thus be made as interesting as previous meetings have been. Secretaries of societies sending delegates should forward the names of their representatives to Dr. Wm. Brodie, of Detroit, who is one of the secretaries of the association.

---

*Mal-praxis.*—In a suit recently brought against Dr. W. B. Gould, of Lockport, N. Y., a verdict was rendered for the defendant. We are glad to notice this return to common sense on the part of juries. From what we learn of the case, any other verdict would have been unjust.

---

With our next number we close Vol. XI. With that issue we shall inclose bills to all indebted to us. As the cheaper plan for the subscriber, and by far the better one for us, we suggest the propriety of advance pay for the coming volume.

